# Disrupting β-Catenin/BCL9 interaction with a peptide prodrug boosts immunotherapy in colorectal cancer

**DOI:** 10.3389/fimmu.2025.1662385

**Published:** 2025-08-29

**Authors:** Peili Wang, Xiao Shang, Jinmei Wang, Weiming You, Shoufei Qu, Yu Yao, Xiaoqiang Zheng

**Affiliations:** ^1^ Department of Hepatology, The Second Affiliated Hospital of Xi’an Jiaotong University, Xi’an, China; ^2^ Department of Medical Oncology, The First Affiliated Hospital of Xi’an Jiaotong University, Xi’an, China; ^3^ Institute for Stem Cell & Regenerative Medicine, The Second Affiliated Hospital of Xi’an Jiaotong University, Xi’an, China; ^4^ National & Local Joint Engineering Research Center of Biodiagnosis and Biotherapy, The Second Affiliated Hospital of Xi’an Jiaotong University, Xi’an, China

**Keywords:** colorectal cancer, peptide, protein-protein interaction, β-catenin, immunotherapy

## Abstract

**Introduction:**

Hyperactivation of the Wnt/β-catenin pathway serves as a central mechanism underlying tumor progression, immune evasion, and resistance to immune checkpoint inhibitor therapy in colorectal cancer (CRC). A pivotal contributor to this process is the binding of β-catenin to B-cell lymphoma 9 (BCL9), which promotes transcription of oncogenes and fosters an immune-suppressive tumor milieu. Consequently, targeting this interaction offers a promising approach to suppress tumor progression and potentiate antitumor immune responses.

**Methods:**

We developed a peptide-based prodrug, Bcl9@TP, designed to competitively bind the BCL9 interface on β-catenin, destabilize the transcriptional complex, and suppress Wnt/β-catenin signaling. Its antitumor efficacy and immune potentiation were assessed *in vitro* using MC38 cells and *in vivo* in murine tumor models.

**Results:**

*In vitro*, Bcl9@TP significantly inhibited MC38 cell proliferation by downregulating β-catenin and its downstream targets, inducing G1-phase cell cycle arrest. *In vivo*, Bcl9@TP treatment markedly reduced tumor burden, with a tumor growth inhibition (TGI) rate of ~62%, significantly higher than the control group. In contrast, anti-PD-1 monotherapy yielded a TGI of only 41%. Notably, combination therapy (Bcl9@TP plus anti-PD-1) produced a more pronounced antitumor effect, with the TGI reaching 82%. Importantly, Bcl9@TP demonstrated favorable systemic biocompatibility and safety.

**Discussion:**

Our findings indicate that disrupting the β-catenin/BCL9 interaction with a peptide-based nanoprodrug represents a compelling strategy to suppress oncogenic signaling and enhance immunotherapy responses in CRC, providing a new angle to boost checkpoint sensitivity.

## 1 Introduction

Colorectal cancer (CRC) ranks among the most prevalent malignancies globally and continues to be a leading cause of cancer-related morbidity and mortality (1, 2). Despite significant advancements in systemic therapies, including targeted drugs and immune checkpoint blockade (ICB), the prognosis for patients with advanced or metastatic CRC remains suboptimal (1, 3–5). The dysregulated activation of the β-catenin signaling cascade is increasingly recognized as a pivotal molecular event contributing to both CRC progression and the development of resistance to immunotherapy (6–8). Within the nucleus, β-catenin forms transcriptional complexes with members of the T-cell factor (TCF)/lymphoid enhancer-binding factor (LEF) family and relies on coactivators such as BCL9 and its homolog BCL9L to enhance oncogenic gene expression (6, 9–11). The activation of downstream effectors, including c-Myc and Cyclin D1, collectively promotes tumor cell proliferation, sustains cancer stem cell properties, facilitates epithelial-mesenchymal transition, drives metastasis, and contributes to immune evasion (10, 12, 13).

A growing body of studies suggests that dysregulation of Wnt/β−catenin signaling fundamentally contributes to the emergence of an immunosuppressive tumor microenvironment (TME) (13). Excessive nuclear β-catenin activation has been shown to impair dendritic cell (DC) recruitment, compromise antigen presentation, inhibit cytotoxic T lymphocyte (CTL) infiltration, and promote the development of an immune “cold” TME, thereby contributing to ICB resistance (14). Excessive nuclear β-catenin activation has been demonstrated to impair DC recruitment, compromise antigen presentation, inhibit CTL infiltration, and foster the development of an immune “cold” TME, thereby contributing to resistance to ICB (15). Additionally, the Wnt/β-catenin pathway influences the polarization of immunosuppressive cells in the TME, particularly tumor-associated macrophages, thereby promoting immune evasion. These findings collectively establish the β-catenin/BCL9 interaction as an attractive therapeutic target with the potential to simultaneously suppress tumor progression (16, 17) and reprogram the CRC immune landscape (18, 19).

While protein–protein interactions (PPIs) have historically been considered “undruggable” due to their intracellular localization and large, flat interfaces, peptide-based therapeutics have emerged as promising tools to selectively disrupt these complexes (20, 21). Compared to small molecules (22, 23), peptide therapeutics offer enhanced target specificity and reduced off-target toxicity, attributed to their distinct spatial configuration and favorable biocompatibility, rendering them a promising candidate for anticancer drug development (24–26). However, the clinical translation of peptide drugs is impeded by inherent challenges such as limited stability, vulnerability to enzymatic degradation, short systemic half-life, and restricted tumor accumulation (27, 28). To overcome these limitations, nanotechnology-based delivery platforms have emerged as a viable solution (29–31). Through structural encapsulation, surface modification, and functional engineering, these platforms can substantially improve peptide stability, extend circulation time, and enhance tumor-targeted accumulation (27, 32, 33). Such strategies have shown significant potential in augmenting therapeutic efficacy in preclinical models of various solid tumors (18, 28, 34).

Here, we present Bcl9@TP, a self-assembling peptide nanoprodrug that selectively disrupts the β-catenin/BCL9 interaction in CRC, delivering direct antitumor activity while augmenting responses to immunotherapy. Bcl9@TP was prepared through a simple, one-pot self-assembly process, forming uniform and stable nanostructures. Mechanistically, Bcl9@TP competitively occupies the BCL9-binding interface on β-catenin, destabilizes the β-catenin/TCF complex, and effectively inhibits downstream Wnt/β-catenin signaling activation. Bcl9@TP displayed robust tumor-suppressive properties when used alone. Moreover, Bcl9@TP reshaped the immunosuppressive TME, further boosting the response to ICIs while maintaining a favorable safety margin throughout treatment. In conclusion, our findings establish Bcl9@TP as a novel, precisely targeted peptide-based nanoprodrug that interferes with the Wnt/β−catenin axis and provides a feasible combination strategy to enhance anticancer immune responses in tumors.

## 2 Materials and methods

### 2.1 Preparation of Bcl9@TP

Bcl9@TP was fabricated through a one−pot self−assembly approach. Initially, the BCL9-targeting peptide (Bcl9-P) was synthesized via Fmoc-based solid-phase peptide synthesis (SPPS) utilizing an automated peptide synthesizer (CS BION336X). Subsequently, 2 mg of the purified BCL9-P was dissolved in a solvent mixture comprising 0.5 mL of anhydrous ethanol and 1.25 mL of ultrapure water. Under magnetic stirring at 70 °C, 1 mL of an aqueous chloroauric acid solution (HAuCl_4_·xH_2_O, 10 mM), 500 μL of thiol-terminated polyethylene glycol amine (NH_2_-PEG-SH, MW 2000 Da, 4 mg/mL), and 2.25 mL of HEPES buffer were sequentially introduced into the peptide solution to form the gold precursor complex. The reaction mixture was subsequently diluted with 2.25 mL of deionized water and an equivalent volume of HEPES buffer, followed by ultrasonication at 40 kHz and 300 W for 10 minutes to facilitate nanoparticle formation. Finally, the crude product underwent purification through dialysis against distilled water, employing a dialysis membrane with a molecular weight cutoff (MWCO, 10 kDa, 4 °C).

### 2.2 Physicochemical characterization

The morphology of Bcl9@TP was visualized by drop-casting the sample onto a carbon-coated copper grid, followed by air drying, and observed using a Talos L120C G2 transmission electron microscope (TEM). High-resolution elemental distribution was assessed by ThermoFisher Talos-F200X scanning TEM. Size characterization of Bcl9@TP nanoparticles was carried out by dynamic light scattering (DLS) on a Zetasizer Nano ZSE.

### 2.3 Animal experiments

All animal procedures were approved by the Biomedical Ethics Committee of Health Science Center of Xi’an Jiaotong University (Numbered 2021-1737). Subcutaneous tumor xenograft models were established by injecting 6 × 10^5^ MC38 cells into C57BL/6 mice. Once tumor reached approximately 100 mm³ (measured daily), the mice were allocated into the following treatment groups (n=5 per group): (1) control (PBS); (2) anti-PD-1(3 mg/kg); (3) Bcl9@TP (2 mg/kg), and (4) Bcl9@TP+anti-PD-1, intravenous (i.v.) injection. Tumor volume (mm³) = (length × width²)/2.

### 2.4 Immunohistochemistry

Tumor tissues were harvested, fixed, embedded in paraffin, and sectioned (4 μm). Then, tissue sections from each treatment group underwent deparaffinization, followed by antigen retrieval in citrate buffer (pH 6.0, at 95 °C). Following this, the sections were blocked and incubated with primary antibodies (overnight, 4 °C). Primary antibodies: anti-Ki-67 (1:400 dilution, CST, 12202), anti-β-catenin (1:200 dilution, Abcam, ab32572), anti-c-Myc (1:200 dilution, Abcam, ab32072), and anti-Cyclin D1(1:200 dilution, CST, 555065). The subsequent day, all sections were washed and treated with the appropriate HRP−conjugated secondary antibody, followed by visualization using a diaminobenzidine (DAB) substrate and counterstaining with hematoxylin. Imaging was conducted using a digital pathology scanner.

### 2.5 Statistical analysis

Statistical evaluation involved an unpaired two-tailed t-test for dual−group analyses, or one-way ANOVA followed by Tukey’s *post hoc* test for multiple cohorts. Numerical outcomes are displayed as mean ± SD, with *, **, ***, and **** marking p−values below 0.05, 0.01, 0.001, and 0.0001.

## 3 Results

### 3.1 Design and one-pot assembly of Bcl9@TP

**Figure 1:**
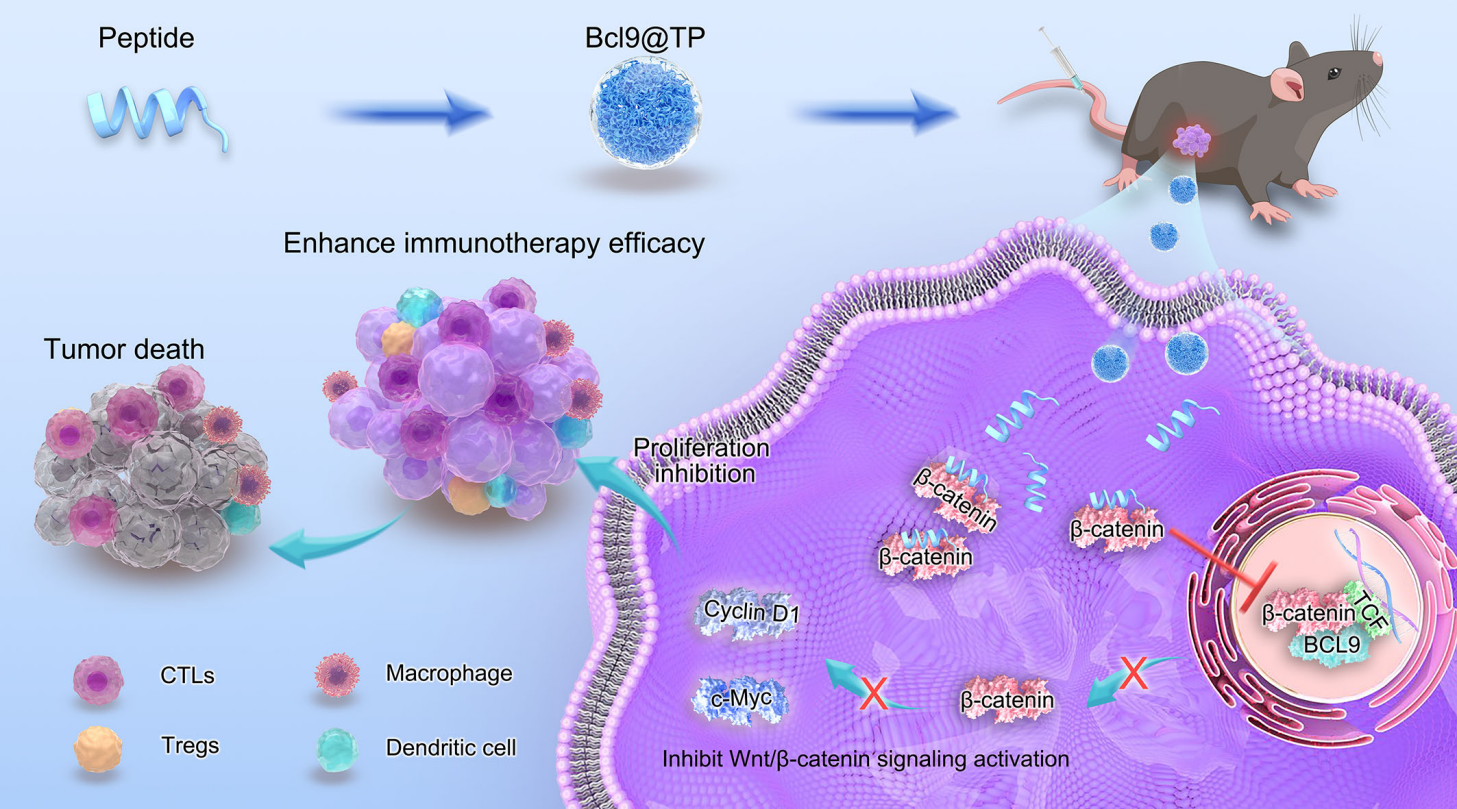
Illustration depicting the construction and therapeutic action of Bcl9@TP nanodrug. By competing for β-catenin binding, it disrupts β-catenin/BCL9 interaction, downregulates oncogenic Wnt signaling, remodels the immunosuppressive TME, suppresses tumor growth, and enhances immune checkpoint inhibitor-mediated antitumor immunity.

Persistent hyperactivation of Wnt signalling is widely recognised as a molecular engine that drives tumour progression and immune escape (6). A principal switch that locks this pathway in perpetual overdrive is the high−affinity complex formed in the nucleus between β−catenin and BCL9 (18). Accordingly, disrupting this interaction has emerged as a compelling strategy for concurrently curbing tumour−intrinsic growth and alleviating the immunosuppressive TME. Capitalizing on this mechanistic paradigm, we converged computational drug design with artificial intelligence-guided screening to generate Bcl9@TP-a high-affinity peptide targeting β-catenin’s Armadillo domain. This self-organizing nanotherapeutic achieves efficient intracellular payload delivery, competitively occluding the β-catenin/BCL9 interface, dismantling β-catenin/TCF complex formation, and attenuating β-catenin-driven transcriptional output. Subsequent downregulation of proto-oncogenic effectors c-Myc and Cyclin D1 yields substantial anti-proliferative effects. Parallel Wnt pathway abrogation reprograms immunosuppressive niches via cytotoxic lymphocyte activation, amplifying anti-PD-1 immunotherapeutic response (**Figure 1**). Our methodology establishes a blueprint for intercepting recalcitrant PPIs and circumventing immunotherapy resistance.

### 3.2 Characterization of Bcl9@TP

**Figure 2:**
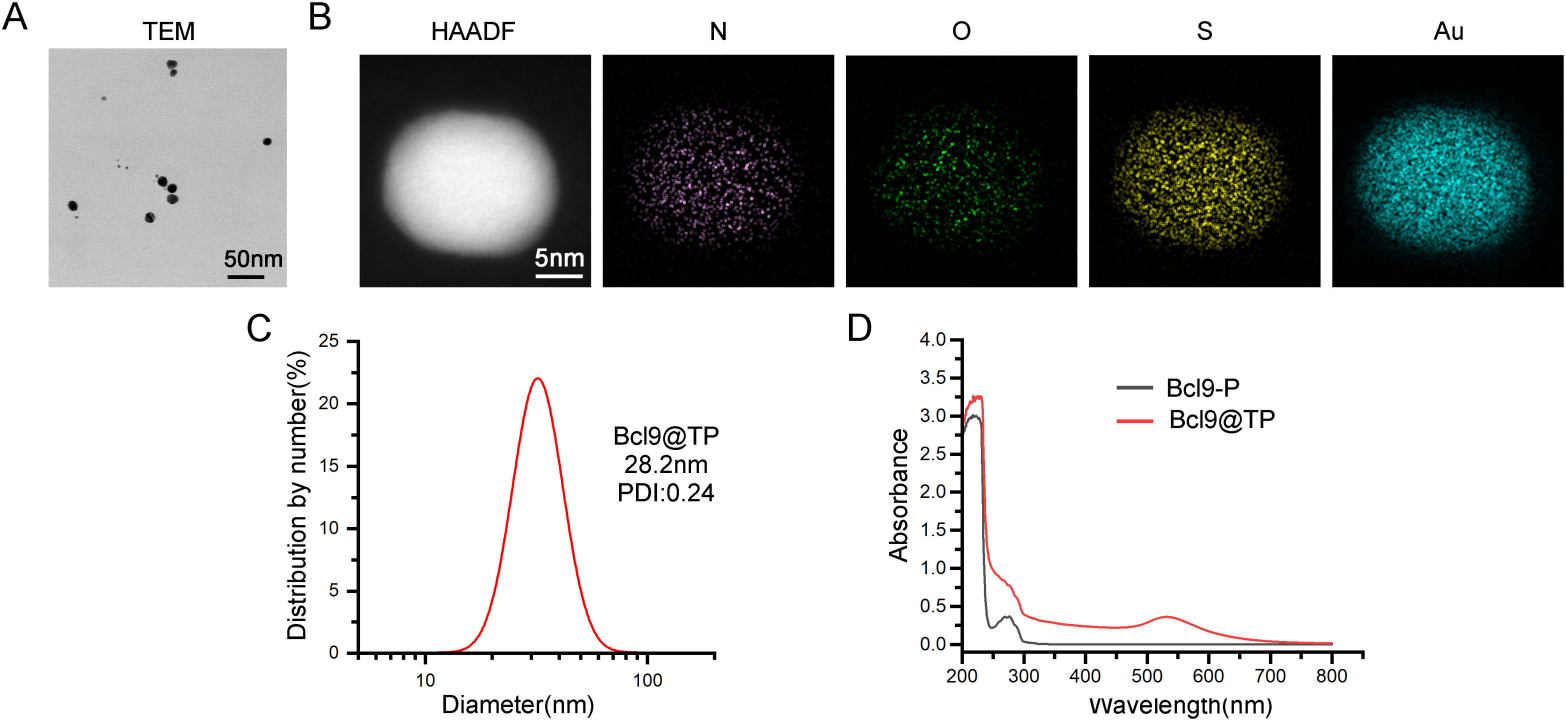
Physicochemical characterization of Bcl9@TP nanoparticles. **(A)** The TEM image showed uniform morphology and stable particle size distribution of Bcl9@TP. **(B)** HAADF-STEM image and elemental mapping confirmed he homogeneous distribution of key components within the nanostructure. **(C)** Characterization of Bcl9@TP nanoparticle size and polydispersity through DLS measurement. **(D)** UV-Vis absorption spectrum validated the successful formation of the Bcl9@TP nanostructure.

Peptide-based nanodrugs have garnered increasing attention in tumor-targeted therapy due to their favorable biocompatibility, precise molecular design, and potential for functional modification. In this study, we developed a novel β-catenin/BCL9-targeting peptide-gold nanoplatform (Bcl9@TP) via a one-pot self-assembly approach, and its physicochemical characteristics were systematically evaluated. TEM revealed that Bcl9@TP nanoparticles exhibited a uniform, spherical morphology with an average diameter, indicating successful nanoparticle formation (**Figure 2A**). Furthermore, high-angle annular dark-field scanning transmission electron microscopy (HAADF-STEM) coupled with elemental mapping revealed the spatial co-distribution of key elements—nitrogen (N), oxygen (O), and sulfur (S), gold (Au)—within the particles, indicating uniform component integration (**Figure 2B**). This elemental distribution pattern is consistent with the expected composition of the peptide-gold hybrid system, demonstrating the molecular uniformity and successful integration of the thiol-modified peptide with gold ions to form a stable nanoconstruct. DLS analysis revealed a mean particle size of roughly 28.2 nm for Bcl9@TP, coupled with a polydispersity index (PDI) of 0.24, indicating excellent colloidal stability and a high degree of uniformity in aqueous media (**Figure 2C**). A characteristic absorption peak at 280 nm was observed via the ultraviolet-visible (UV–Vis) spectrum, attributed to the amide bonds within the peptide backbone. That serves as indirect evidence for successful peptide assembly and structural integrity (**Figure 2D**). Furthermore, the cellular uptake efficiency of Bcl9@TP was quantitatively evaluated using flow cytometry (**Supplementary Figure S1**). After 6 hours of co-incubation with MC38 cells, the internalization rate of FITC-labeled Bcl9-P was merely 7.6%, whereas Bcl9@TP demonstrated a markedly higher uptake rate of 96%. Collectively, the characterization results demonstrate that the Bcl9@TP nano-prodrug has a uniform particle size, optimal dispersibility, and enhanced membrane permeability, providing a robust basis for evaluating its biological effects.

### 3.3 Bcl9@TP inhibits cell proliferation via suppressing Wnt/β-catenin signaling

**Figure 3:**
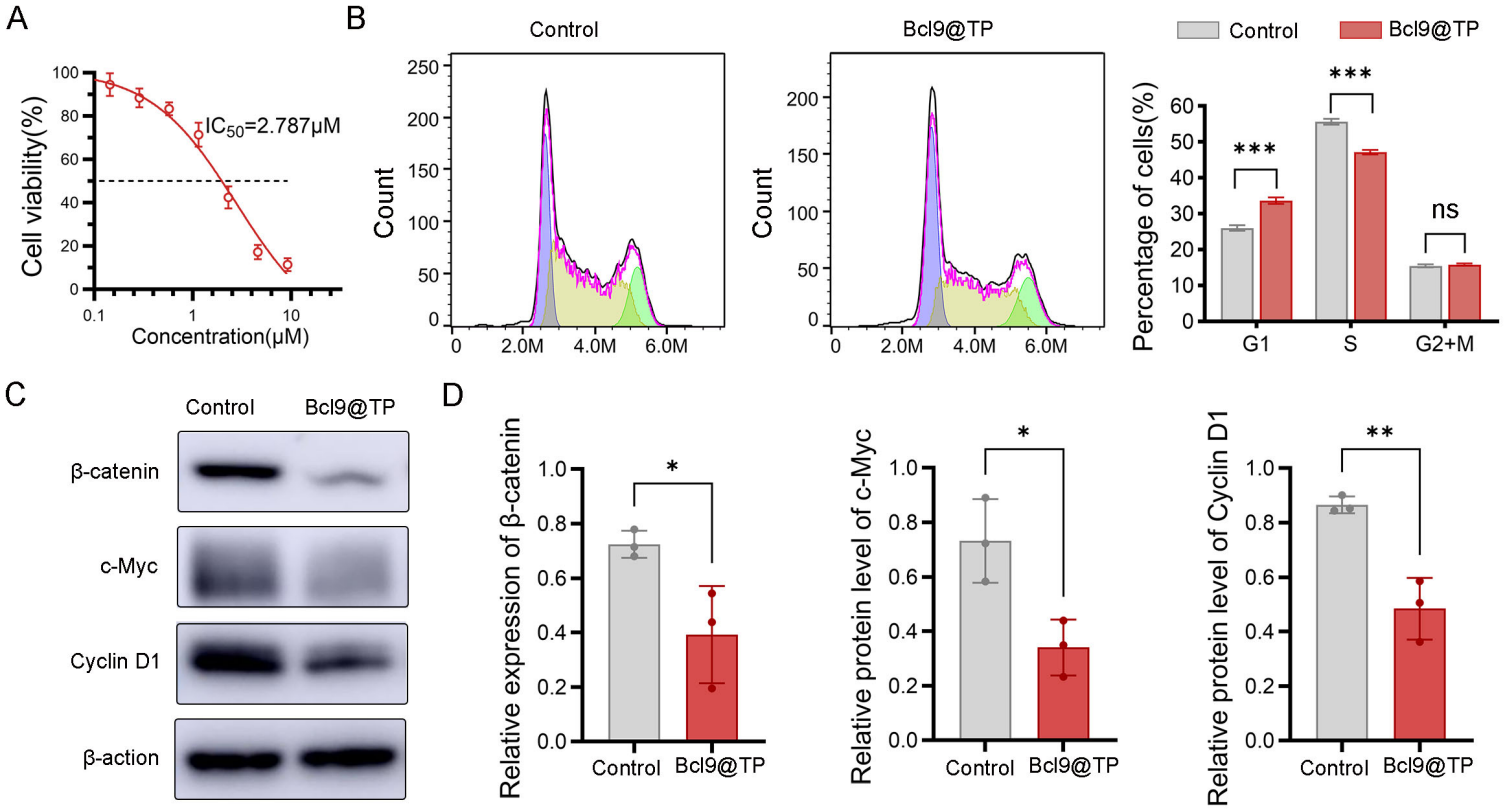
*In vitro* antitumor activity and mechanistic studies of Bcl9@TP. **(A)** Cell viability of MC38 cells exposed to a range of Bcl9@TP concentrations *via* CCK-8 assay (n=3). **(B)** Flow cytometry analysis of cell cycle distribution after Bcl9@TP treatment, indicating significant G1 phase arrest (n=3). **(C, D)** Western blot image **(C)** and quantification analysis **(D)** demonstrated Bcl9@TP-induced downregulation of β-catenin, c-Myc, and Cyclin D1 in MC38 cells (n = 3). unpaired t-test, *p < 0.05; **p < 0.01; ***p < 0.001.

We engineered the Bcl9@TP nanoplatform to selectively compete with BCL9 for binding to β-catenin and subsequently evaluated its antitumor efficacy *in vitro*. Cellular viability assessment demonstrated concentration-dependent cytotoxicity in MC38 cells treated with escalating Bcl9@TP doses (48-hour exposure), yielding a half-maximal inhibitory concentration of 2.8 μM (**Figure 3A**), confirming potent proliferation suppression. Flow cytometric cell cycle profiling (24-hour treatment) revealed substantial G1 phase accumulation concurrent with S-phase depletion versus untreated controls (**Figure 3B**), signifying effective G1 arrest induction and replicative blockade. Moreover, Western blotting was conducted to examine the molecular changes associated with Wnt/β-catenin pathway suppression. Bcl9@TP treatment led to an approximately 45% reduction in β-catenin protein levels relative to controls, along with notable downregulation of key downstream oncogenic targets c-Myc and Cyclin D1 by 53% and 44%, respectively (**Figure 3C**). These findings indicate that Bcl9@TP competitively binds to the β-catenin/BCL9 interface, destabilizes the transcriptional complex, and suppresses oncogene expression, collectively contributing to its anti-tumor effect.

### 3.4 *In vivo* anti-tumor and immunotherapy sensitization efficacy

**Figure 4:**
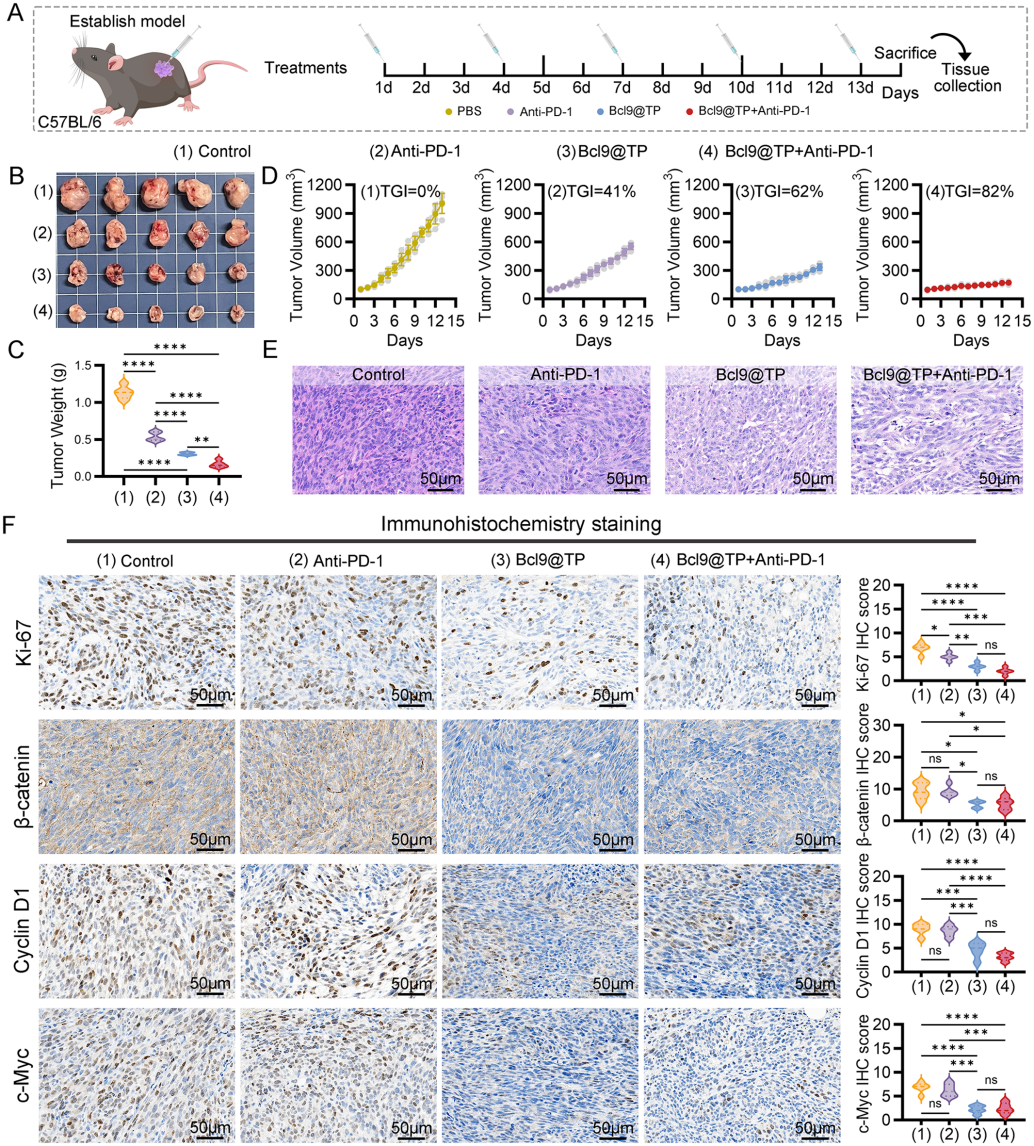
*In vivo* antitumor efficacy of Bcl9@TP in a subcutaneous CRC tumor model. **(A)** Illustration depicting the procedure for establishing the subcutaneous tumor xenograft model in mice, along with the timeline of drug administration. **(B)** Representative ex vivo images of tumor tissues harvested from mice receiving different therapeutic interventions (n = 5). **(C)** Comparative analysis of tumor weights among various treatment groups at the study endpoint (n = 5). **(D)** Tumor growth curves reflecting the effects of different treatments (n = 5). **(E)** Representative H&E-stained tumor tissues from each group, scale bars:50 μm. **(F)** IHC assessment and relative expression analysis of Ki-67, β-catenin, c-Myc, and Cyclin D1. (n = 5), scale bars:50 μm. Significance: one-way ANOVA, *p < 0.05; **p < 0.01; ***p < 0.001; ****p < 0.0001.

To determine whether Bcl9@TP can effectively suppress Wnt/β-catenin-driven tumor progression and immune evasion, we investigated the therapeutic potential *in vivo* using an immunocompetent subcutaneous tumor model. Female C57BL/6 mice bearing established MC38 tumors were randomly assigned to different groups: 1) control, 2) anti-PD-1, 3) Bcl9@TP, and 4) combination of Bcl9@TP with anti-PD-1(**Figure 4A**). Tumor growth was monitored throughout the study, and final tumor volumes were recorded to evaluate therapeutic efficacy. Following euthanasia, subcutaneous tumors were excised from mice in each treatment group and photographed for gross morphological comparison (**Figure 4B**). Tumor weight measurements revealed a marked reduction in tumor burden in both the Bcl9@TP alone group and combination (Bcl9@TP + anti-PD-1) group compared to the control and anti-PD-1 groups, with the combination group exhibiting the most pronounced effect—an approximate 85% decrease in tumor mass (**Figure 4C**). Consistently, tumor growth curves mirrored these findings (**Figure 4D**). A tumor growth inhibition rate (TGI) of ~62% was attained with Bcl9@TP, reflecting its significant therapeutic activity. Anti-PD-1 treatment alone demonstrated moderate antitumor activity, with a TGI of around 41%. Notably, the combination of Bcl9@TP with PD−1 antibody exhibited superior antitumour efficacy, achieving the highest TGI (~82%) among all treatment groups, thereby underscoring the considerable potential of this strategy to enhance ICB therapy. Histopathological assessment of H&E-stained tumor sections demonstrated that the control group displayed densely packed tumor cells with high mitotic activity, indicating aggressive tumor growth. In contrast, the combination treatment exhibited sparse tumor cell distribution (**Figure 4E**). These data underscore the therapeutic potential of Bcl9@TP not only as a monotherapy but also as a potent sensitizer that enhances the efficacy of ICB.

To further elucidate the mechanistic basis of Bcl9@TP’s antitumor activity, we conducted IHC analyses on tumor sections from each treatment group. Staining for the proliferation marker Ki-67 revealed a significant reduction in tumor cell proliferation following Bcl9@TP monotherapy, with the Ki-67 index decreasing by approximately 55% compared to the control group. Notably, combining Bcl9@TP with anti-PD-1 therapy led to a more pronounced suppression of proliferative activity, with an approximate 70% reduction in Ki-67 positivity, consistent with the observed tumor growth inhibition (**Figure 4F**). Mechanistically, IHC staining demonstrated markedly reduced expression of β-catenin in tumors from the Bcl9@TP and combination therapy groups, showing approximately 44% and 42% reductions, respectively, relative to the Con and anti-PD-1 monotherapy groups. This suppression is attributable to Bcl9@TP’s ability to antagonise the β−catenin–BCL9 association, which interferes with β-catenin nuclear translocation and transcriptional activation. The expression of key Wnt/β-catenin downstream oncogenic targets, including Cyclin D1 and c-Myc, was significantly downregulated in the Bcl9@TP treatment groups, by approximately 49% and 67%, respectively. The combination therapy group observed a similar downward trend (**Figure 4F**). These findings confirm the effective *in vivo* blockade of β-catenin signaling by Bcl9@TP.

To investigate the immunomodulatory effects of Bcl9@TP on the TME, we performed immunofluorescence staining on tumor tissues from each treatment group (**Supplementary Figure S2**). Bcl9@TP monotherapy significantly increased CD3^+^CD8^+^ cytotoxic T lymphocyte (CTL) infiltration within TME, showing a 3-fold increase relative to controls. This effect was further amplified by co-administration with anti–PD-1, resulting in a 4.8-fold increase relative to control and approximately 2.2-fold that of anti–PD-1 monotherapy. Conversely, compared to controls, Bcl9@TP markedly reduced the proportion of CD4^+^FOXP3^+^ regulatory T cells (Tregs) by around 53%, while the combination regimen led to a more pronounced reduction of 74% (P < 0.01). These findings indicate that Bcl9@TP promotes robust CTL-mediated antitumor immunity and attenuates Treg-driven immunosuppression, with combination therapy yielding superior synergistic immunomodulatory effects.

Overall, the results reveal that Bcl9@TP effectively disrupts the β-catenin/BCL9 interaction within the TME, leading to the destabilization of transcriptional co-activator complexes and subsequent suppression of oncogenic and proliferative gene expression. Notably, Bcl9@TP markedly potentiated the therapeutic efficacy of ICIs, highlighting its promise as a combinatorial strategy to overcome immune resistance and enhance antitumor immunity in colorectal cancer.

### 3.5 *In vivo* biosafety assessment

**Figure 5:**
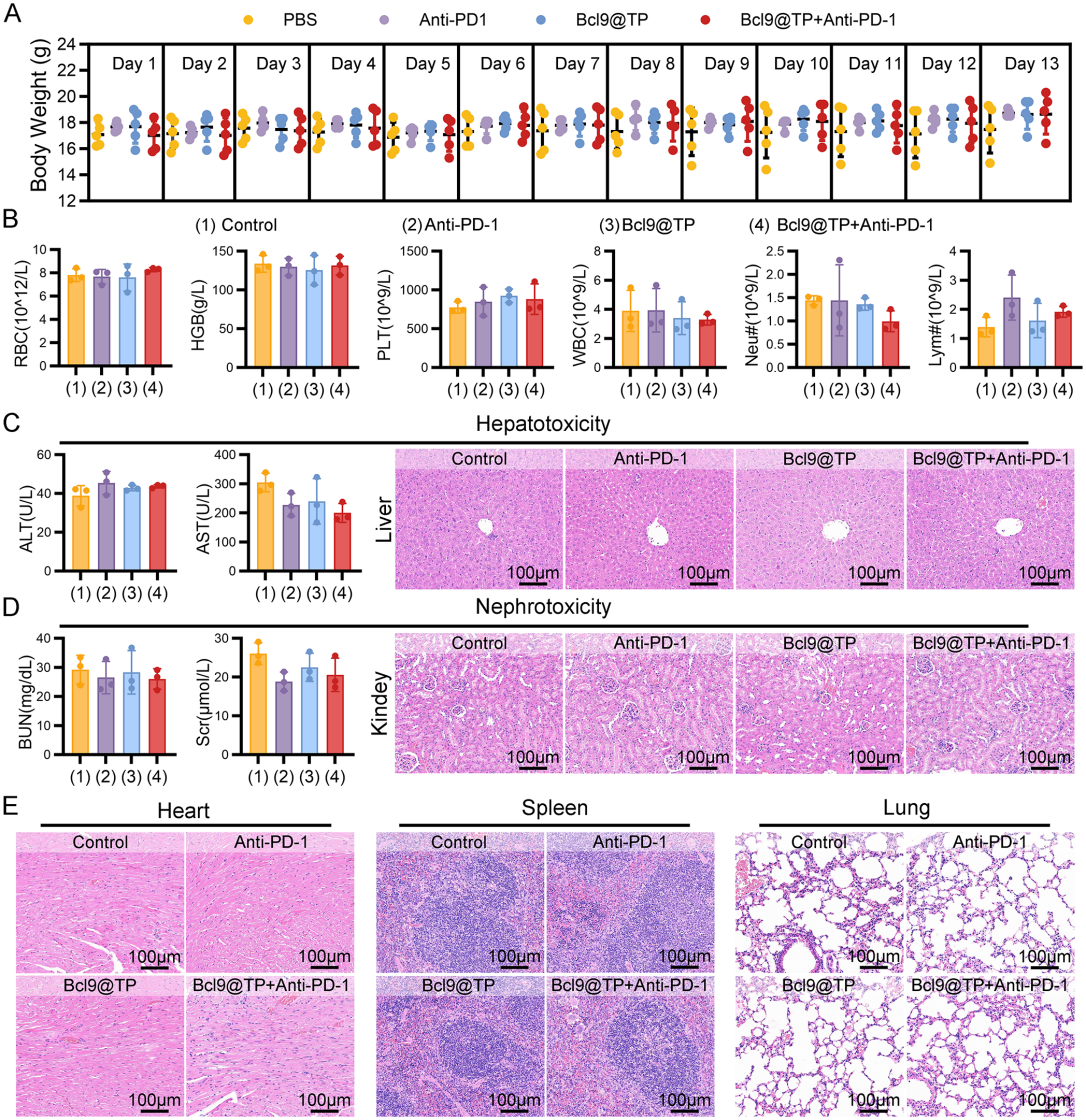
Biosafety assessment of Bcl9@TP *in vivo*. **(A)** Body weight monitoring of mice during treatment showed no significant systemic toxicity (n=5). **(B)** Quantitative blood profiling of mouse groups post−therapy, including red cell (RBC), hemoglobin (HGB), platelet (PLT), white cell (WBC), as well as neutrophil (Neu^#^) and lymphocyte (Lym^#^) (n = 3). **(C)** Hepatotoxicity assessment in mice from different treatment groups, including serum biochemical analysis of ALT and AST levels (n = 3) and histopathological evaluation of liver tissue by H&E staining. **(D)** Nephrotoxicity assessment in mice from different treatment groups, comprising serum measurements of BUN and Scr levels (n = 3) and histological examination of renal tissue using H&E staining. **(E)** H&E staining of cardiac, splenic, and pulmonary tissues under varying treatments (scale bar: 100 μm).

The biosafety of Bcl9@TP is a critical prerequisite for its clinical translation, particularly when applied in combination with immunotherapies such as PD-1 checkpoint blockade. Multiple physiological and histopathological parameters were monitored throughout the treatment period to assess systemic toxicity. The dynamic body weight monitoring data demonstrated a progressive upward trend in the Bcl9@TP monotherapy group throughout the treatment course. Notably, even the combination therapy group showed no significant reduction in body weight. (**Figure 5A**). Furthermore, detailed hematological analyses were performed at the end of the treatment cycle. Bcl9@TP, both as monotherapy and in combination with anti–PD−1, did not alter key hematological metrics (such as red blood cells, white blood cells, and platelets) relative to controls, underscoring its lack of detectable blood-related toxicity (**Figure 5B**).

Serum biochemical assessments revealed that alanine aminotransferase (ALT) and aspartate aminotransferase (AST) remained in the normal ranges across all groups, with no statistically significant differences observed. Correspondingly, hematoxylin and eosin (H&E) staining of liver sections revealed no signs of inflammatory cell infiltration, hepatocellular necrosis, or architectural disruption, suggesting the absence of hepatotoxicity (**Figure 5C**). Regarding renal function, blood urea nitrogen (BUN) and serum creatinine (Scr) levels were comparable among all groups. Histological evaluation of kidney sections demonstrated intact glomerular structures and normal renal tubules without degeneration or inflammation, indicating no apparent nephrotoxicity (**Figure 5D**). Further histopathological assessments of the heart, spleen, and lung based on H&E staining showed no evidence of inflammatory infiltration, parenchymal injury, or structural disorganization, effectively excluding potential toxic effects of Bcl9@TP on these organs (**Figure 5E**). Collectively, these findings demonstrate the strong systemic biosafety of Bcl9@TP, supporting its suitability for repeated intravenous administration and its compatibility with immune checkpoint therapies. The favorable safety profile provides a strong foundation for the further development of Bcl9@TP as a clinically relevant nanoplatform for colorectal cancer treatment.

## 4 Discussion

The dysregulated Wnt/β-catenin cascade constitutes a defining pathological feature in CRC, orchestrating both intrinsic oncogenic processes and extrinsic immunosuppressive reprogramming within the tumor milieu (35). Central to this mechanism is the persistent nuclear localization of β-catenin, which actively subverts immune surveillance through three interconnected pathways: 1) inhibition of DC recruitment, 2) exclusion of CTL (36), and 3) induction of immunologically quiescent tumor phenotypes (15, 37). This triad of immune dysfunction culminates in the evolution of checkpoint blockade-resistant malignancies. Consequently, therapeutic disruption of the β-catenin-BCL9 protein interaction interface represents a dual-pronged intervention strategy (17) – simultaneously impeding malignant progression while reconditioning the immunosuppressive TME. Despite compelling preclinical validation, the clinical translation of agents targeting this signaling axis continues to confront substantial translational barriers.

Peptide-based PPI inhibitors offer a promising solution to overcome the limitations of conventional small-molecule drugs (22, 38, 39), as peptides exhibit superior structural adaptability and surface complementarity, enabling them to selectively disrupt large (21, 32), flat PPI interfaces such as β-catenin/BCL9. Moreover, advances in computational design and phage display screening have facilitated the discovery of novel peptide scaffolds capable of engaging previously “undruggable” intracellular targets. However, the clinical application of peptide therapeutics is hindered by several intrinsic drawbacks, including poor enzymatic stability, limited plasma half-life, inefficient intracellular delivery, and inadequate tumor targeting (27). These challenges are particularly pronounced for PPIs involving nuclear-localized targets, where successful intervention requires both efficient cellular uptake and precise subcellular distribution (28).

To address these challenges, various delivery strategies have been developed, including the use of cell-penetrating peptides, pH-responsive nanocarriers, redox-sensitive conjugates, and tumor-targeting ligands to enhance endosomal escape and nuclear localization (40–43). In parallel, biomimetic systems such as exosome-mimetic vesicles (44, 45) or erythrocyte membrane-coated nanoparticles (46, 47) have shown promise in prolonging circulation time and reducing off-target immunogenicity. These strategies provide critical pharmacokinetic advantages for peptide therapeutics by improving their plasma half-life, minimizing premature degradation, and promoting tumor targeting via the enhanced permeability and retention effect (28, 48). Overcoming these obstacles is pivotal for fully exploiting the therapeutic advantages of peptide-based PPI-targeting agents in cancer treatment (39).

In this study, we developed an intelligent nanodelivery system to address these limitations by constructing the peptide-based nanoprodrug Bcl9@TP via a one-pot self-assembly method (49). This design yielded uniform, biocompatible nanoparticles with enhanced structural stability, prolonged systemic circulation, and efficient tumor accumulation. Mechanistic studies confirmed that Bcl9@TP competitively disrupts β-catenin/BCL9 interactions, destabilizes the β-catenin/TCF transcriptional complex, and inhibits aberrant Wnt signaling activation, thereby suppressing tumor growth. Importantly, Bcl9@TP not only exhibited potent antitumor efficacy as a monotherapy but also effectively remodeled the TME by enhancing T cell infiltration and function, leading to a significant synergistic effect with PD-1 blockade. These results provide proof-of-concept for peptide-based PPI inhibitors as viable candidates to overcome Wnt-driven immune resistance in CRC.

Looking ahead, further optimization of peptide design—such as incorporating unnatural amino acids, cyclization strategies, or stapling technologies—may improve proteolytic stability and intracellular retention. Additionally, next-generation nanocarriers incorporating stimuli-responsive release mechanisms, tumor-penetrating peptides, or immune cell-mediated delivery could further enhance specificity and bioavailability. Importantly, expanding the evaluation of Bcl9@TP into genetically engineered or patient-derived CRC models with varying Wnt activity levels will be critical to validate its broad therapeutic applicability. Integrating molecular profiling of Wnt/β-catenin pathway status, immunophenotyping of the TME, and identification of predictive biomarkers could also facilitate precision-guided clinical translation and patient stratification.

In conclusion, this study presents a nanotechnology-enabled strategy to overcome longstanding barriers in peptide drug delivery and demonstrates the feasibility of targeting β-catenin/BCL9 interactions to suppress tumor progression and enhance immunotherapy responsiveness. Moving forward, further optimization of peptide design, exploration of next-generation nanocarriers, and comprehensive evaluation across diverse tumor models are warranted. Additionally, integrating Wnt pathway activity profiling, TME characterization, and biomarker-guided patient stratification may facilitate clinical translation and maximize therapeutic benefit. Collectively, our findings establish a robust foundation for the development of PPI-targeted nanomedicine and offer new avenues to improve outcomes in CRC and other Wnt-driven malignancies.

## Data Availability

The original contributions presented in the study are included in the article/**Supplementary Material**. Further inquiries can be directed to the corresponding authors.
