# Supplementary material for: Disrupting β-Catenin/BCL9 interaction with a peptide prodrug boosts immunotherapy in colorectal cancer
Source: Front Immunol. 2025 Aug 29;16:1662385. doi: 10.3389/fimmu.2025.1662385 (PMC12425980; doi:10.3389/fimmu.2025.1662385)
Supplement: Supplementary file 1 [file DataSheet1.docx]

Supplementary Material

Disrupting β-Catenin/BCL9 Interaction with a Peptide Prodrug Boosts Immunotherapy in Colorectal Cancer

Peili Wang^1^, Xiao Shang^3^, Jinmei Wang^1,2^, Weiming You^1,4^, Yu Yao^3,*^, Xiaoqiang Zheng^1,2*^

^1^ Department of Hepatology, The Second Affiliated Hospital of Xi’an Jiaotong University, Xi’an 710004, China

^2^Institute for Stem Cell & Regenerative Medicine, The Second Affiliated Hospital of Xi’an Jiaotong University, Xi’an 710004, China

^3^Department of Medical Oncology, The First Affiliated Hospital of Xi'an Jiaotong University, Xi’an 710061, China

^4^National & Local Joint Engineering Research Center of Biodiagnosis and Biotherapy, The Second Affiliated Hospital of Xi’an Jiaotong University, Xi’an 710004, China.

**Correspondence:**

Corresponding Author: Yu Yao， Xiaoqiang Zheng

yaoyu123@xjtufh.edu.cn，[zhengxiaoqiang@xjtu.edu.cn](mailto:zhengxiaoqiang@xjtu.edu.cn)

# 1 Supplementary Figures


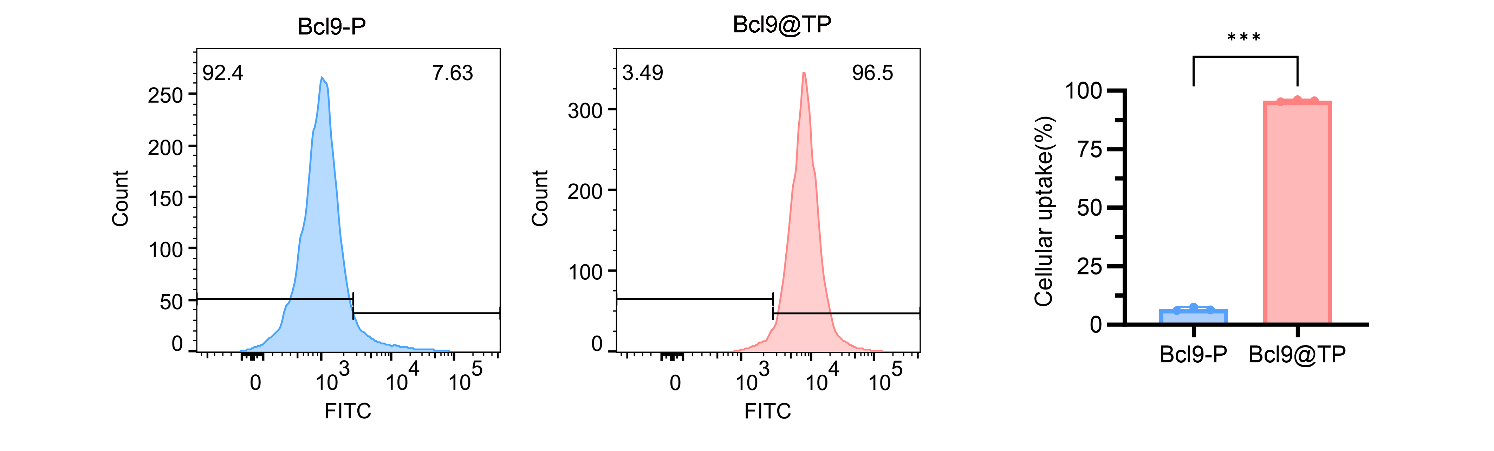
**Figure S1.** Quantitative analysis of FITC-labeled Bcl9-P and Bcl9@TP internalization by MC38 cells after 6 h incubation was performed using flow cytometry. Data are presented as mean ± standard deviation (n=3). ***p<0.001 (Student’s t-test).


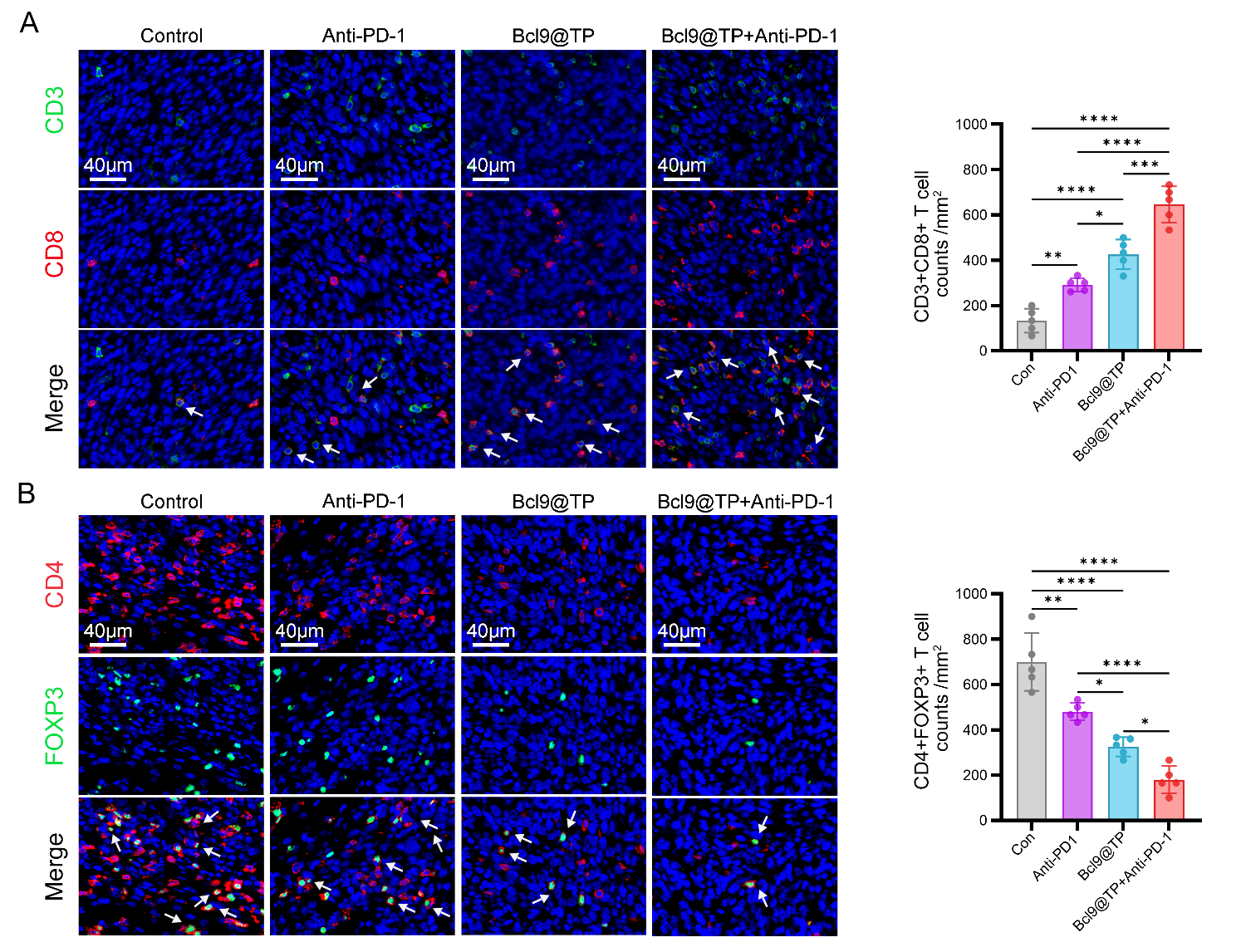


**Figure S2.** (A) Fluorescence images and quantitative analysis showing the distribution of CD3⁺CD8⁺ T cells in tumor tissues across different treatment groups (green: CD3, red: CD8, blue: DAPI nuclear staining). (B) Fluorescence images and quantitative analysis demonstrating the infiltration ratio of CD4⁺FOXP3⁺ regulatory T cells (Tregs) in tumor tissues among different treatment groups (red: CD4, green: FOXP3, blue: DAPI). White arrows indicate double-positive cells. Scale bar: 40 μm.

# 2 Supplementary Material and Methods

## 2.1 Cell Culture and Cytotoxicity Assay

MC38 cells (mouse colon adenocarcinoma) were cultured in high-glucose DMEM supplemented with 10% fetal bovine serum (FBS) under standard conditions (5% CO₂, 37°C). The cells were obtained from the Cell Bank, Chinese Academy of Sciences, Shanghai.

In the exponential growth phase, MC38 cells were plated into 96-well plates (3 × 10³ cells per well) and treated with varying concentrations of Bcl9@TP for 48 hours. Afterward, the medium was replaced with 100 μL of fresh medium containing 10% CCK-8 reagent. After incubation, the absorbance at 450 nm was measured and recorded using a microplate reader, followed by data analysis.

## 2.2 Cellular Uptake

Exponentially growing MC38 cells were incubated with FITC-labeled Bcl9@TP or FITC-labeled Bcl9‑P for 6 hours. Following incubation, cells were harvested, washed thoroughly with PBS, and subjected to flow cytometric analysis to quantify intracellular FITC fluorescence, thereby evaluating cellular uptake efficiency.

## 2.3 Cell Cycle Analysis

MC38 cells were first cultured to the logarithmic growth phase and then treated with BCL9@TP for 24 hours. After treatment, the cells were collected, washed with cold PBS, and fixed overnight in 70% pre-cooled ethanol. Following fixation, cells were centrifuged, washed, and stained with PI in the dark. Cell cycle arrest was analyzed by comparing the treatment groups using a NovoCyte flow cytometer.

## 2.4 Western Blot

First, after BCL9@TP treatment, the cells were lysed using RIPA buffer (containing protease inhibitors, 1mM) to extract total protein. The protein concentration of each sample was measured. Equal amounts of denatured protein were then separated by SDS-PAGE electrophoresis and transferred onto polyvinylidene fluoride (PVDF) membranes. After transfer, the membrane was blocked and incubated with primary antibodies (overnight at 4°C). After multiple washes, the membrane was incubated with secondary antibodies for 1 hour at RT. Finally, protein bands were detected using chemiluminescence (ECL), and densitometric analysis of the bands was performed using image analysis software for protein quantification. Primary antibodies: β-catenin (Abcam, ab32572), Cyclin D1 (CST, 555065), c-Myc (Abcam, ab32072), dilution 1:1000, and β-actin (Proteintech, 66009-1-Ig, dilution 1:8000) as the internal control.

## 2.5 Immunofluorescence staining

Tissue sections were deparaffinized, rehydrated through a graded ethanol series, and subjected to antigen retrieval using citrate buffer. After blocking with BSA, primary antibodies were applied and incubated overnight at 4 °C. The following day, HRP-conjugated secondary antibodies were added and incubated for 50 minutes at room temperature, followed by tyramide signal amplification (TSA) and TBST washes. Then, the above steps were repeated for additional targets. Nuclei were counterstained with DAPI. Fluorescence images were acquired using the Pannoramic MIDI digital slide scanner. Primary antibodies: CD3 (servicebio, GB15014), CD8 (servicebio, GB15068), CD4 (abcam, ab183685), FOXP3 (servicebio, GB112324).

## 2.6 Safety Evaluation

Peripheral whole blood and serum samples were collected from treated mice for hematological and biochemical analyses to assess potential hematotoxicity, hepatotoxicity, and nephrotoxicity. Major organs, including the heart, liver, spleen, lungs, and kidneys, were harvested, fixed in 4% paraformaldehyde, embedded in paraffin, sectioned, and subjected to hematoxylin and eosin (H&E) staining. Histopathological examination was performed to evaluate the extent of tissue injury in each treatment group.
